# Functional Surfaces for Passive Fungal Proliferation Control: Effect of Surface Micro- and Nanotopography, Material, and Wetting Properties

**DOI:** 10.1021/acsabm.4c00387

**Published:** 2024-07-01

**Authors:** Vasiliki Tselepi, Panagiotis Sarkiris, Dimitrios Nioras, Erminta Tsouko, Dimitrios Sarris, Evangelos Gogolides, Kosmas Ellinas

**Affiliations:** † Laboratory of Advanced Functional Materials and Nanotechnology, Department of Food Science and Nutrition, School of the Environment, University of the Aegean, Leoforos Dimokratias 66, Myrina 81400, Lemnos, Greece; ‡ Institute of Nanoscience and Nanotechnology NCSR “Demokritos”, Aghia Paraskevi 15341, Attiki, Greece; § Laboratory of Physico-Chemical and Biotechnological Valorization of Food Byproducts, Department of Food Science & Nutrition, School of Environment, University of the Aegean, Leoforos Dimokratias 66, Myrina 81400, Lemnos, Greece; ∥ Theoretical and Physical Chemistry Institute, National Hellenic Research Foundation, 48 Vassileos Constantinou Ave., 11635 Athens, Greece

**Keywords:** antifungal surfaces, Aspergillus awamori, superhydrophobic surfaces, superhydrophilic surfaces, plasma treatment, chemical etching, fungal proliferation control

## Abstract

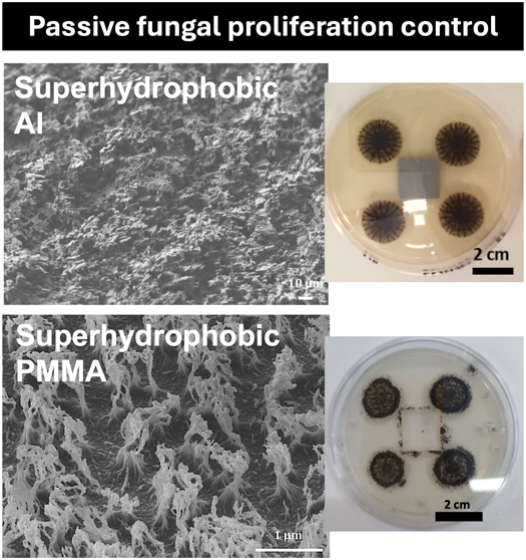

Fungal proliferation can lead to adverse effects for human health, due to the production of pathogenic and allergenic toxins and also through the creation of fungal biofilms on sensitive surfaces (i.e., medical equipment). On top of that, food spoilage from fungal activity is a major issue, with food losses exceeding 30% annually. In this study, the effect of the surface micro- and nanotopography, material (aluminum, Al, and poly(methyl methacrylate), PMMA), and wettability against *Aspergillus awamori* is investigated. The fungal activity is monitored using dynamic conditions by immersing the surfaces inside fungal spore-containing suspensions and measuring the fungal biomass growth, while the surfaces with the optimum antifungal properties are also evaluated by placing them near spore suspensions of *A. awamori* on agar plates. Al- and PMMA-based superhydrophobic surfaces demonstrate a passive-like antifungal profile, and the fungal growth is significantly reduced (1.6–2.2 times lower biomass). On the other hand, superhydrophilic PMMA surfaces enhance fungal proliferation, resulting in a 2.6 times higher fungal total dry weight. In addition, superhydrophobic surfaces of both materials exhibit antifouling and antiadhesive properties, whereas both superhydrophobic surfaces also create an “inhibition” zone against the growth of *A. awamori* when tested on agar plates.

## 1 Introduction

Fungi hold significant ecological, agricultural, and biotechnological value. There are around 5 million species on Earth after extensive evolution, with about 300 identified as pathogens, endophytes, saprobionts, or epiphytes for a variety of hosts in terrestrial and aquatic habitats.^1−3^ Fungi can contaminate air flow and air filtration systems, hospital facilities, buildings, ships, and food products.^4^ They colonize under different environmental conditions due to their adaptability on various surfaces, while their growth can be favored at 25–30 °C, in moist environments. The Food and Agriculture Organization (FAO) reports that 1/3 of the global food production annually results in waste or losses.^5^ The metabolic products of fungi such as organic acids, e.g., gluconic, citric, and oxalic acid, result in food contamination, and furthermore, these bioproducts can cause corrosion of the materials, especially of metals and their alloys, leading to severe corrosion failures in stone monuments, buildings, automobiles, ships, and aircrafts.^6^ It is also impressive that species of *Aspergillus* have been reported to secrete organic acids capable of hydrolyzing powdered stone and chelate minerals and further convert them into glucose-based media, which underscore the metabolic versatility of fungi and their capacity to exploit diverse environmental resources for growth and survival.^7^ In order to limit the proliferation of the fungi in as many environments as possible, many early and accurate pathogen detection methods have been proposed.^8^ However, along with successful detection, it is also vital to prevent pathogen (i.e., fungi) attachment and proliferation. To do so, advanced materials and surfaces with appropriate topography, wetting, and material properties should be realized.

Superhydrophobic surfaces enable liquids to move on them with reduced friction, which can be translated to low adhesion and interactions with liquids on such surfaces.^9−12^ Therefore, it is expected that wetting control can either significantly reduce the adhesion of microorganisms and remove any humidity, which favors microbial growth (superhydrophobic state), or promote it (superhydrophilic state).^13^ To this end, some first research efforts have been presented in the literature. For example, Lee and Hwang developed a micro/nanostructured, superhydrophobic coating for a brazed aluminum heat exchanger (BAHE).^14^ The coating enables self-cleaning from the developed fungus *Penicillium implicatum* with a small amount of water. Kim and Hwang reported the extent of fungal growth on superhydrophobic, superhydrophilic, weakly hydrophobic, and hydrophobic aluminum surfaces, chemically treated, using three common airborne fungi: *P. implicatum*, *Cladosporium cladosporioides*, and *Aspergillus fumigatus*.^15^ They implemented a direct (on the surface) and an indirect (near the surface) protocol and showed that superhydrophobic surfaces can prevent the adhesion and spread of fungal contamination on the air conditioning evaporator. In other work, a thin superhydrophobic film was used to coat goose feathers that serve as an insulator material.^16^ The coating involved a thin superhydrophobic layer created by organosilicon precursors, specifically, hexamethyldisiloxane (HMDSO) and hexamethyl disilazane (HMDSN), using plasma chemical gas-phase deposition, and the authors reported that the coating exhibited high resistance against the fungal species *Aspergillus flavus*, *Aspergillus niger*, and *A. fumigatus*.

Examples of artificial surfaces to fight other microorganisms (i.e., bacteria) have also been reported in the literature.^17,18^ In most cases, the studies deal with the incorporation of a biocide agent usually in the form of particles or nanoparticles incorporated in paints or coatings,^7,19,20^ but recently, biomimetic approaches taking advantage of the beneficial role of nanoscale topography in the antibacterial action and studies related to passive and “green” concepts (without biocides) have been proposed.^13,21−24^

However, most of the studies on passive approaches that address microorganisms’ proliferation on surfaces have been conducted with bacteria. Very few studies have investigated the proliferation of fungi, while reports on the effect of different factors, such as surface topography, surface chemistry, or the choice of material on fungal growth, are scarce. Herein, we investigated the effect and the possible synergy among surface micro/nanotopography, wettability, and material type on the proliferation control of *Aspergillus awamori* (a relatively understudied fungal food pathogen), using two different protocols; the first is done under dynamic conditions by immersing the target surfaces inside a fungal spore suspension, while in the second, which can be termed as indirect, surfaces were also evaluated by placing them and spore suspensions on agar plates (conditions which favor fungal proliferation). It is demonstrated that superhydrophobic surfaces of both materials tested (Al and PMMA) significantly reduced the production of fungal biomass total dry weight compared to untreated surfaces (0.3 mg instead of 0.65 mg for the untreated Al surfaces and 0.68 mg instead of 1.11 mg for the untreated PMMA surfaces), acting as passive antifungal surfaces (1.6–2.2 times lower fungal biomass). On the contrary, when the other extreme wetting state (superhydrophilicity) was realized, superhydrophilic PMMA surfaces significantly promoted fungal proliferation, resulting in a 2.6 times higher biomass (2.9 mg total dry weight instead of 1.11 mg for the untreated PMMA surfaces). In addition, superhydrophobic surfaces of both materials showed excellent antifouling and antiadhesive properties, whereas superhydrophobic surfaces and particularly those made from aluminum created an “inhibition” zone against the growth of *A. awamori* when tested on agar plates. It is therefore demonstrated that on-demand and passive fungal proliferation control can be achieved after careful design of the surface wetting, material, and micro/nanotopography properties.

## 2 Experimental Section

### 2.1 Materials

Aluminum (alloy 1015) and PMMA (IRPEN, Spain) surfaces with dimensions of 20 mm × 20 mm × 2 mm were used as specimens. For both materials, untreated (flat) and micro/nanotextured surfaces were used as described below. The selection of these commonly used materials (i.e., aluminum (Al) and poly(methyl methacrylate) (PMMA)) was made because both have been extensively studied in the past and, therefore, the fabrication methods as well as their performance could be reproducible and repeatable from others.

### 2.2 Surface Micro/Nanostructuring and Chemistry Modification

For the preparation of the hierarchical (micro/nano)-structured Al samples, a simple and commonly used two-step wet etching method was used; this method can be easily repeated by others, and it has been shown in our previous work to achieve a superhydrophobic state; at least 7 min of wet etching in HCl is required to create microscale features on Al, whereas longer durations can provide deeper structures appropriate for superhydrophobicity.^25^ In more detail, the aluminum surfaces were first immersed into a 9.25% v/v aqueous solution of hydrochloric acid for 12 min to create a first-step microscale topography on the aluminum substrate. Then, nanotopography was created on top of the microstructures by immersing the microtextured surfaces inside boiling water following the bohemitage process, which is a really environmental friendly wet method (only water is used).^26^ Following this two-step wetting method, the hierarchical micro/nanostructured surfaces were coated with a hydrophobic film of ∼30 nm through C_4_F_8_ plasma deposition inside an inductively coupled plasma (ICP) reactor (plasma deposition conditions: 900 W power, 25 sccm gas flow rate, and 40 mTorr pressure for 1 min) to render them superhydrophobic or were used as superhydrophilic without the addition of the extra coating.

For the PMMA surfaces, plasma micro- and nanotexturing was used. Plasma micro/nanotexturing is a powerful method that can be used in every organic polymer and transform it to superhydrophobic featuring water static contact angles (WSCA) greater than 150° and contact angle hysteresis lower than 10°, regardless of its initial nature. In addition, the plasma method we use is a two-step, dry, green, and environmentally friendly approach. The superhydrophilic surfaces were created after treatment with oxygen plasma, using a custom-built inductively coupled plasma (ICP) reactor, for 1 and 6 min (plasma deposition conditions: 300 W power, 250 V bias voltage, 100 sccm gas flow rate, and 6 mTorr pressure). The application of the bias voltage during oxygen plasma treatment leads to anisotropic etching of the polymeric sample. During etching, a very small number of etching inhibitors (usually less than 1%) from the electrode are sputtered on the surface, acting as micro- and nanomasks resulting in nanograss–nanofilament formation, which grows to larger hierarchical micro/nanostructures as etching time increases. More details about the process can be found in some previous work,^27,28^ but the process is highly reproducible, and it has been implemented for the fabrication of micro/nanotextured surfaces focused on a plethora of applications (i.e., atmospheric water collection,^29^ antifogging surfaces,^30^ repellency of low-surface tension liquids,^27^ direct covalent immobilization of protein molecules on organic polymers,^31^ functional microdevices,^32,33^ etc.). In order for the PMMA surfaces to become superhydrophobic, after the plasma micro/nanotexturing step for 1 or 6 min, a thin hydrophobic coating (about 30 nm) was deposited using plasma deposition of C_4_F_8_ gas for 1 min (plasma deposition conditions: 900 W power, 25 sccm gas flow rate, and 40 mTorr pressure).

### 2.3 Surface Morphology and Wetting Property Characterization

The characterization of the morphology induced on the surfaces was done by scanning electron microscopy using a JEOL JSM-7401F FEG, at 2 kV beam voltage. For the wetting property characterization, both the water static contact angle and the contact angle hysteresis were measured using a Kruss DSA 30 system. For the water static contact angle measurements, 5 μL of deionized water drops was used and the average value as well as the standard deviation of three measurements was calculated. For the contact angle hysteresis measurement, a 5 μL drop of deionized water is placed on the surface, the volume of the droplet is increased up to 25 μL, and during the increase of the droplet volume, the advancing contact angle is being measured; likewise, the receding contact angle is being measured during the decrease of the droplet volume. The difference between the advancing and receding contact angles is the contact angle hysteresis (CAH).

### 2.4 Microorganism Maintenance and Preculture Conditions

The fungal strain *A. awamori* EXF-213 (Nakaz. 1907) was kindly provided by the Infrastructural Mycosmo Centre and Microbial Culture Collection Ex (University of Ljubljana, Slovenia). *Aspergillus* is one of the three fungal strains (the other two are *Fusarium* and *Penicillium*) that can spoil food by the production of mycotoxins.^34^ The strain was maintained in slopes at 4 °C, containing 5% (w/v) wheat milling byproducts and 1.5% (w/v) agar. To prepare a more concentrated spore suspension, *A. awamori* spores were freshly prepared in 250 mL Erlenmeyer flasks (250) containing solid medium, similar to the substrate used in the slopes. A volume of 10 mL of deionized water and Tween 80 (0.01%, v/v) was aseptically added into each slope, and the surface of the slope was scratched with a wire loop. Subsequently, 500 μL of this spore suspension was added on the surface of the solid media of flasks. They were incubated at 30 °C for 3–4 days. Fungal spore suspensions were prepared by adding 100 mL of deionized water (supplemented with Tween 80, 0.01%, v/v) followed by vigorous shaking using glass beads of 4 mm diameter. A spore suspension of ∼2–3 × 10^7^ spores/mL was obtained, which was further properly diluted with deionized water to achieve a final spore suspension of ∼0.5–0.8 × 10^6^ spores/mL.

All materials and solutions used were previously autoclaved at 121 °C for 15 min. All processes were performed under sterile conditions by using a laminar flow cabinet (BIOBASE, Biological Safety Cabinet, BSC 1100IIA2-X).

### 2.5 Fungal Proliferation Assessment Protocol

For the fungal proliferation assessment, we applied two protocols. In the first, dynamic protocol, the modified surfaces were placed into the center of 6-well tissue culture plates (growth area: 9.5 cm^3^, media volume: 1.9–2.9 mL) in sterile conditions. Subsequently, 2 mL of *A. awamori* spore suspension (see Section 2.4) was added in each plate followed by incubation for 2 weeks, at 30 °C, in static incubators (LabTech, Daihan Labtech Co., LTD). Fungal biomass of each well was recovered after 2 weeks by centrifugation (9000 rpm, 5 °C, 15 min), dried at 85 °C for 24 h, and cooled in a desiccator to obtain the fungal biomass total dry weight (TDW). For both types of material surfaces (Al and PMMA), fungal proliferation was studied in three independent experiments, with three replications for each surface.

In the second one, which is termed as “indirect”, spore suspensions were placed away from the surfaces on agar, and the effect of the surfaces on the fungal proliferation was studied. In more detail, aluminum and PMMA surfaces (untreated and superhydrophobic) were placed on sterile PDA (Condalab, Laboratorios Canada S.A., final pH at 25 °C: 5.6 ± 0.2) Petri dishes, and each surface was placed on the agar (center). Spore suspensions with a concentration of 10^7^ spores/mL and a volume of 10 μL were inoculated on four spots around the square-shaped Al surfaces (untreated and superhydrophobic), each one placed 1 cm away from the surface edge. These samples were placed inside a static incubator (LabTech, Daihan Labtech Co., LTD) under conditions that favor fungal growth (30 °C). Fungal proliferation was monitored by capturing images every 2 days for a total duration of 7 days. Then, images were analyzed using ImageJ software to accurately measure the distance of the fungal colony from the edge of the surfaces. Four measurements in each sample were made (one for each inoculation point), and the average value of the distance, as well as its standard deviation, was calculated.

Finally, the antifouling and antiadhesion performance of the superhydrophobic Al and PMMA surfaces was also evaluated by washing the samples with water followed by optical microscopy observation (Olympus optical microscope CX21).

## 3 Results and Discussion

### 3.1 Surface Morphology and Wetting Property Characterization

**Figure 1:**
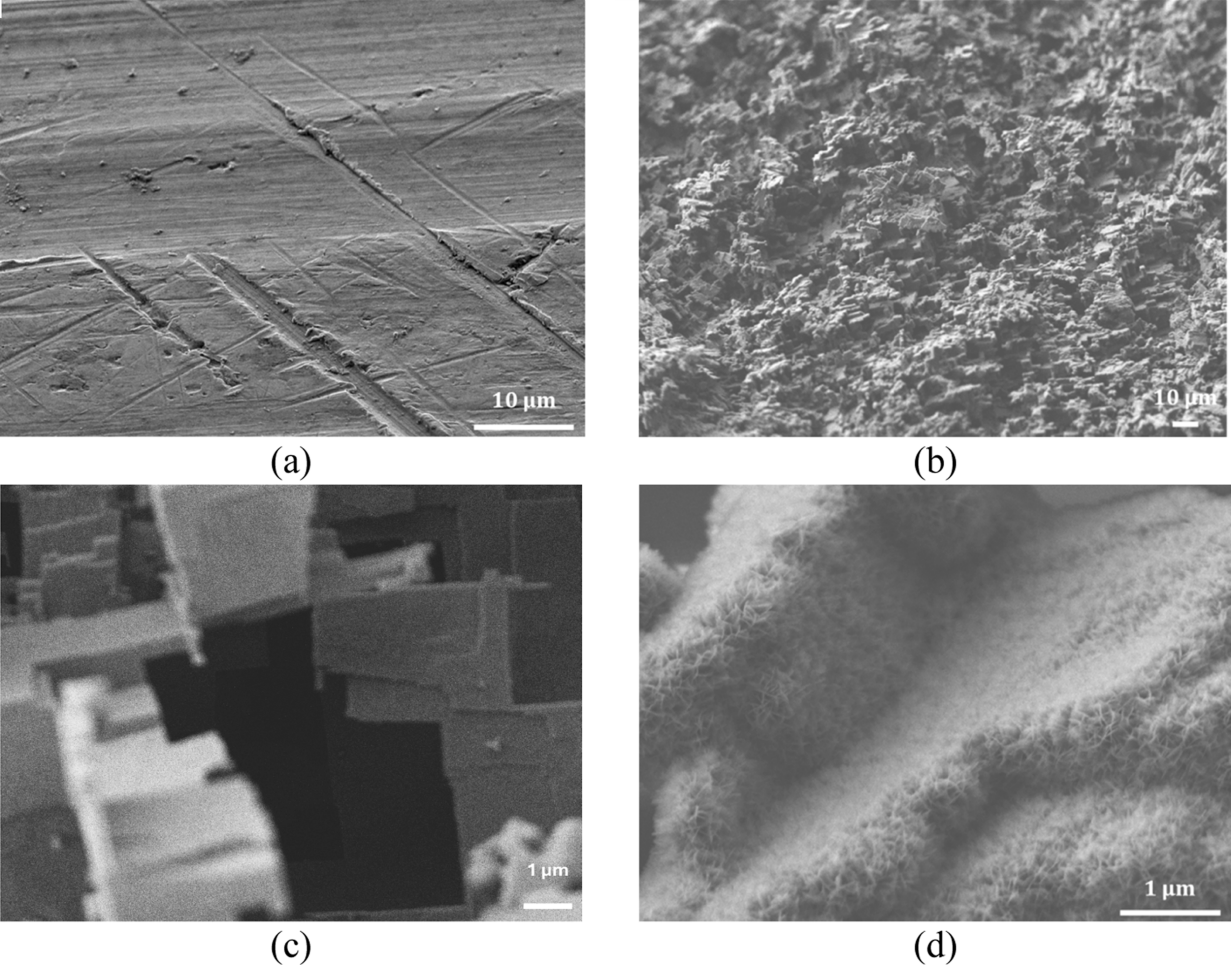
SEM images of the Al surfaces (a) untreated (at 2.000× magnification), (b) microstructured (at 500× magnification), (c) microstructured (at 10.000× magnification), and (d) microstructured and nanostructured (at 20.000× magnification). Images are tilted by 45°.

Both materials (Al and PMMA) were roughened, and their surface morphology was characterized with scanning electron microscopy (SEM). Figure 1 shows the SEM images of the Al surfaces before (Figure 1a) and after the creation of microstructures (Figure 1b,c) as well as the micro- and nanostructures (Figure 1d) using the two-step wet etching process described in Section 2.2. The microscale features created in Al are extremely high (height >20 μm, average width >5 μm) as we have shown in our recent work,^25^ in which Fourier analysis of SEM images of Al surfaces was performed.

Table 1 shows the wetting properties of untreated and treated aluminum surfaces. Untreated aluminum exhibits an intermediate wetting behavior (water static contact angle: 74°). The untreated aluminum surface becomes hydrophobic with the water static contact angle exceeding 110° and high contact angle hysteresis >20°, if a thin hydrophobic layer is deposited by C_4_F_8_ plasma deposition. For the realization of the superhydrophilic Al surface, wet etching using HCl is combined with water boiling (bohemitage process), and Al becomes superhydrophilic due to the formation of micro- and nanoscale features (Figure 1d). In order to make it superhydrophobic, a thin hydrophobic layer, by means of C_4_F_8_ plasma deposition, is applied on the superhydrophilic Al surfaces. Superhydrophobic Al surfaces exhibit a high water static contact angle (>170°) and low contact angle hysteresis (∼2°).

**Table 1:** Water Static Contact Angle (WSCA), Advancing Contact Angle (ACA), Receding Contact Angle (RCA), and Contact Angle Hysteresis (CAH) Measurements for the Al Surfaces^a^

types of surfaces	WSCA (deg)	ACA (deg)	RCA (deg)	CAH (deg)
untreated Al	74 ± 1°	N/A	N/A	N/A
hydrophobic flat Al	110 ± 2°	132 ± 1°	110 ± 2°	22°
12 min hierarchical (micro/nano) superhydrophilic Al	<10°	N/A	N/A	N/A
12 min hierarchical (micro/nano) superhydrophobic Al	171 ± 2°	172 ± 1°	170 ± 2°	2 ± 1°

a N/A stands for not applicable.

**Figure 2:**
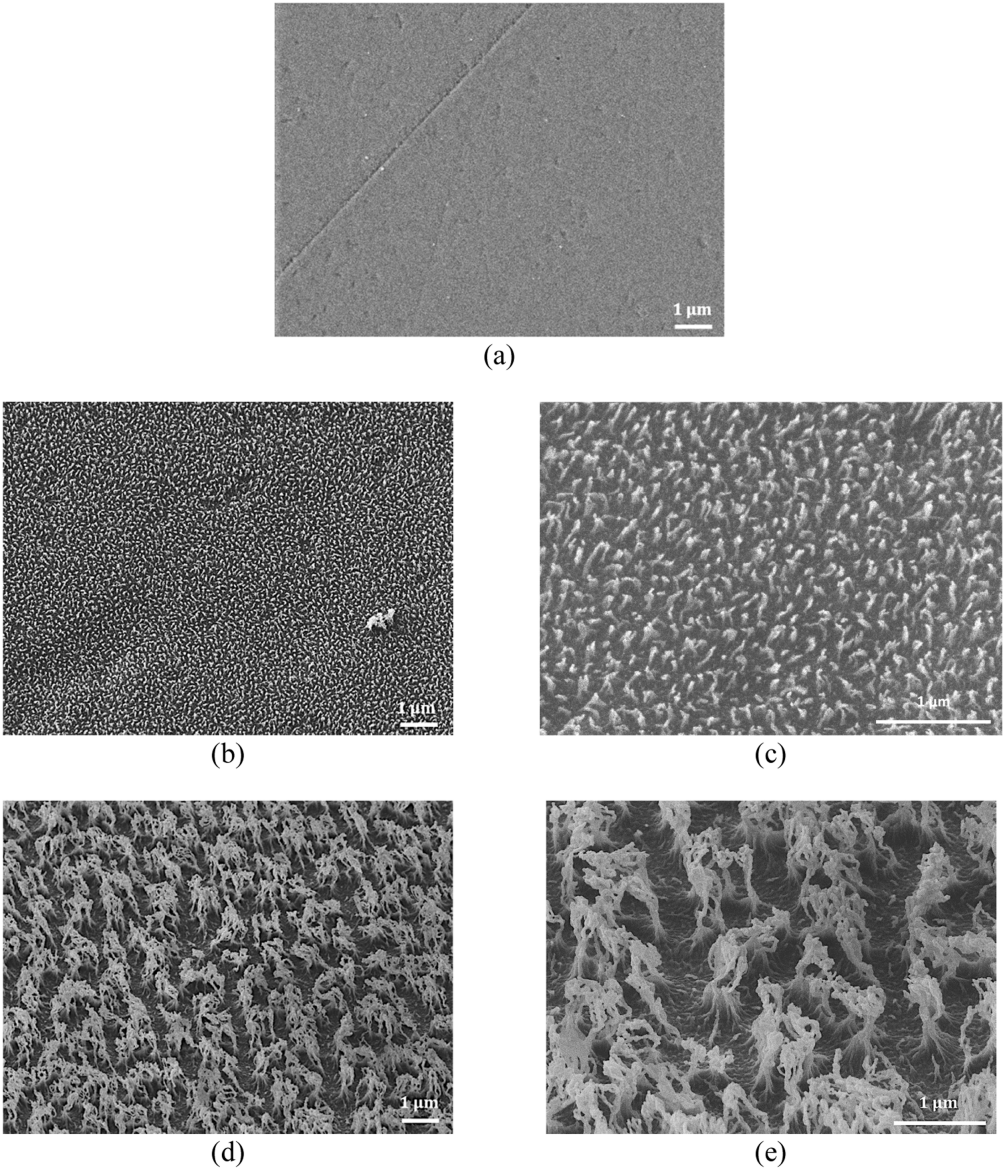
SEM images of the PMMA surfaces (a) before treatment (at 10.000× magnification) and after 1 min of O_2_ plasma etching (b) at 10.000× magnification and (c) at 25.000× magnification and after 6 min of O_2_ plasma etching (d) at 10.000× magnification and (e) at 25.000× magnification. Images are tilted by 45°.

In Figure 2, the SEM images of the oxygen (O_2_) plasma-treated surfaces are shown. It is clear that after O_2_ etching for 1 min, dense nanofilament formation takes place (filament height <500 nm), which gradually grow and bundle together in larger (height >1 μm) multiscale (micro- and nanoscale) structures as etching time increases (6 min). However, in PMMA, the topography features are significantly smaller compared to the features of textured Al. The wetting properties of the PMMA surfaces are controlled over roughness and proper surface chemistry, through the oxygen plasma micro/nanotexturing step. The oxygen plasma step creates functional groups such as C=O, COOH, and OH groups; thus, the surfaces after the plasma step will be hydrophilic and superhydrophilic. In previous work, it has been shown that the longer the treatment of the plasma step, the higher the content of functional groups.^31^ On the other hand, by adding a hydrophobic layer, the surface chemistry changes and CF*_x_* groups are created.

Again, WSCA and CAH measurements show that the topographies created after plasma etching in combination with the deposition of a hydrophobic coating provide several wetting states: hydrophilic (1 min of O_2_ plasma etching), superhydrophilic (6 min of O_2_ plasma etching), superhydrophobic with high hysteresis (1 min of O_2_ + hydrophobic coating), and superhydrophobic surfaces (6 min of O_2_ + hydrophobic coating). Table 2 presents the wetting properties of all of the different PMMA surfaces fabricated.

**Table 2:** Water Static Contact Angle (WSCA), Advancing Contact Angle (ACA), Receding Contact Angle (RCA), and Contact Angle Hysteresis (CAH) Measurements for the PMMA Surfaces^a^

types of surfaces	WSCA (deg)	ACA (deg)	RCA (deg)	CAH (deg)
untreated PMMA	65 ± 1°	N/A	N/A	N/A
hydrophobic flat PMMA	110 ± 2°	117	87	30 ± 2°
superhydrophobic 1 min (O_2_ plasma + hydrophobic coating)	150 ± 2°	160	145	15 ± 2°
superhydrophobic 6 min (O_2_ plasma + hydrophobic coating)	160 ± 2°	161	159	2 ± 1°
hydrophilic 1 min O_2_ plasma	25 ± 2°	N/A	N/A	N/A
superhydrophilic 6 min O_2_ plasma	3 ± 1°	N/A	N/A	N/A

a N/A stands for not applicable.

### 3.2 Fungal Proliferation Control: The Effect of Surface Micro- and Nanotopography, Material, and Wetting Properties

**Figure 3:**
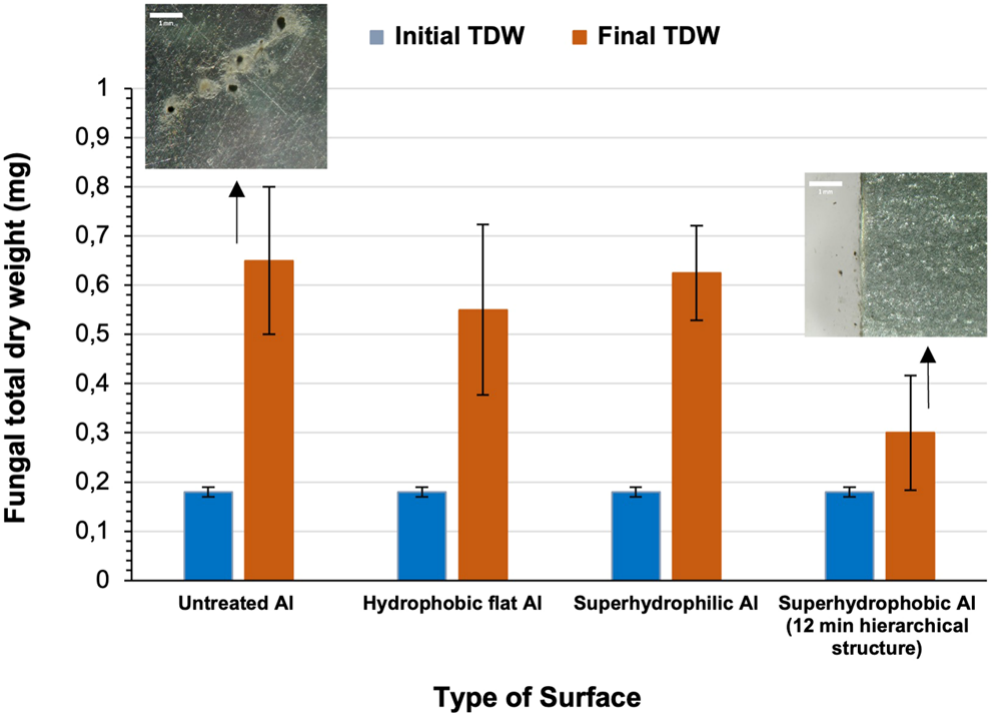
Fungal total dry weight (TDW) obtained from the untreated Al surface, hydrophobic flat Al surface, superhydrophilic Al surface, and 12 min hierarchical superhydrophobic Al surface. Initial fungal biomass TDW is 0.19 mg. Representative images of the fungal proliferation on the surfaces are also shown. On superhydrophobic Al surfaces, no fungal spores are observed.

Four types of Al surfaces are used in the fungal proliferation study: untreated surfaces, hydrophobic flat surfaces, 12 min wet-etched hierarchical superhydrophilic surfaces, and 12 min wet-etched hierarchical superhydrophobic surfaces. The initial spore suspension’s total dry weight (TDW) used to evaluate the properties of Al is 0.19 ± 0.1 mg. After 2 weeks of incubation, the total fungal dry weight is measured for all aluminum surfaces and compared to the initial value in order to investigate the effect of each surface in fungal proliferation (Figure 3).

After 2 weeks of incubation, the TDW for the untreated surfaces is 0.65 mg; it is 0.63 mg for the superhydrophilic, 0.55 mg for the superhydrophobic with high hysteresis (hydrophobic flat), and 0.30 mg for the superhydrophobic. It is evident that fungal proliferation in the culture plate containing the superhydrophobic surface with the hierarchical topography is 2.2 times lower from the weight of the biomass when using the untreated slightly hydrophilic surfaces, in which fungal biomass becomes 3.6 times higher compared to the initial biomass. It is therefore evident that the fungal growth on superhydrophobic Al practically ceased and the fungus development has been significantly delayed. This is also evident by the images captured during the evaluation, which are shown in Supporting Information Section S1. This effect can be attributed to (a) the microscale as well as nanoscale topography features which in combination with the low surface energy and chemistry of the hydrophobic coating (mainly containing CF_*x*_ groups coming from the plasma processing gas C_4_F_8_) prohibit the fungal spores from attaching and growing on them (Supporting Information Figure S1) and (b) the material properties of Al, which will be discussed in detail later in this section.

The low adhesion behavior of superhydrophobic Al compared to the untreated and superhydrophilic Al is also evident in the images provided in Supporting Information Section S2. Superhydrophilic surfaces exhibit high adhesion (low WSCA and high CAH) and increased surface roughness, which is expected to increase the available surface area for fungal growth with the development of hyphae and biofilms. However, the results presented in Figure 3 show that the fungal TDW on superhydrophilic surfaces (0.63 mg) is slightly lower than that on the untreated surfaces (0.65 mg). This is possibly due to the deep surface features (micro/nanosurface topography) after wet etching, which in combination with the surface material (Al) may enable mechanical “fungicidal” effects instead of promoting proliferation (The same observation has been reported by others for bacteria).^21^ In particular, it is possible that the presence of high and multiscale topography features in combination with the material properties possibly disrupts the formation of stable fungal biofilms or alters the distribution of nutrients and signaling molecules essential for fungal growth, contributing to the observed decrease in TDW. Thus, the potential benefits of increased surface area, which are expected to promote fungal proliferation, are outweighed. Moreover, the wetting behavior of the surface can further modulate fungal adhesion and growth. In the case of the superhydrophilic Al surface, the rapid spreading of water across the surface may create a barrier that inhibits fungal attachment and colonization. The capillary action of water within the rough surface structure could prevent fungal spores from establishing firm contact with the substrate, limiting their ability to proliferate effectively.

**Figure 4:**
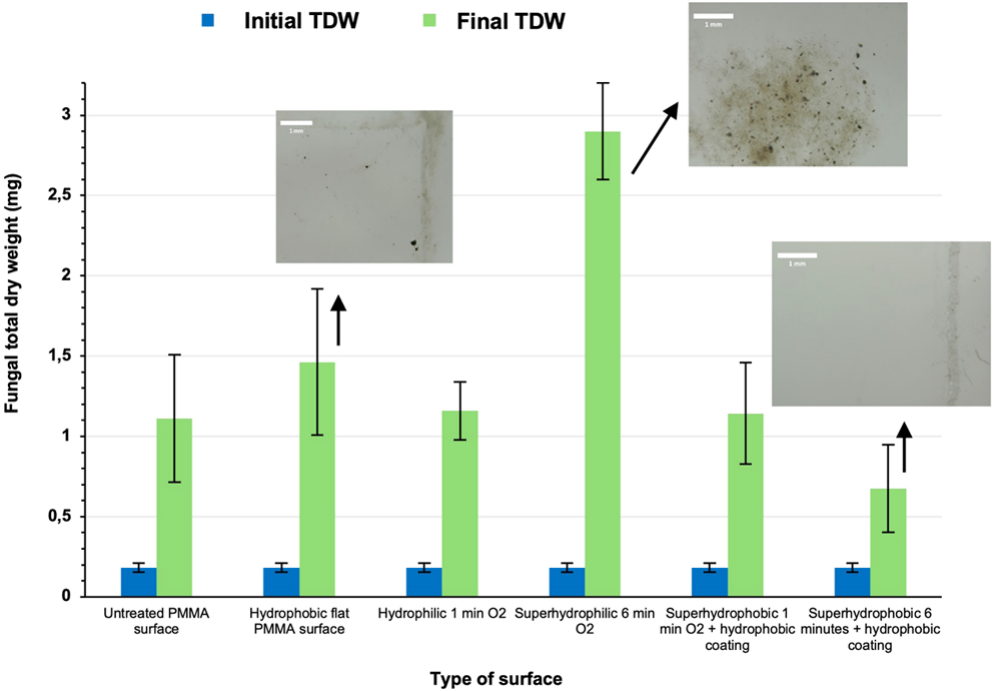
Fungal total dry weight (TDW) obtained from untreated PMMA surfaces, hydrophobic flat PMMA surfaces, hydrophilic 1 min O_2_ plasma surfaces, superhydrophilic 6 min O_2_ plasma surfaces, superhydrophobic with high hysteresis 1 min (O_2_ plasma + hydrophobic coating) surfaces, and superhydrophobic 6 min (O_2_ plasma + hydrophobic coating) surfaces. Initial fungal TDW is 0.17 mg. Representative images of the fungal proliferation on the surfaces are also shown.

In PMMA surfaces, plasma micro/nanotexturing enables the realization of more types of surfaces since the topography geometry and scale can be controlled by controlling the etching processing parameters. Thus, in PMMA surfaces, fungal growth is studied on six types of PMMA surfaces, which are named as untreated, flat hydrophobic, hydrophilic, superhydrophilic, superhydrophobic with relatively high hysteresis, and superhydrophobic surfaces with low hysteresis. In this study, the initial TDW spore suspension biomass was 0.18 ± 0.1 mg. Figure 4 shows that for the untreated surfaces, the final fungal biomass TDW is 1.11 mg; for the flat hydrophobic surfaces with a thin C_4_F_8_ coating, it is 1.46 mg, for the hydrophilic surfaces, it is 1.16 mg, and for the superhydrophilic surfaces, it is 2.9 mg. On the other hand, the fungal biomass TDW for the 1 min O_2_ superhydrophobic surface was found to be 1.14 mg and for the 6 min O_2_ superhydrophobic, it is 0.75 mg.

Similar to that observed with Al, the lowest TDW values are recorded for the 6 min plasma micro/nanotextured superhydrophobic surfaces which exhibit hierarchical micro- and nanoscale topography and the lowest CAH and thus adhesion. It is also observed that 1 min superhydrophobic surfaces with the higher hysteresis (15°) and significantly smaller, less complex, yet denser filament-like topography (filament height <500 nm) are not affecting fungal proliferation, as the TDW for the untreated PMMA is 1.11 mg and for the 1 min superhydrophobic PMMA surface with high hysteresis, TDW is 1.14 mg. It is therefore clear that the topography scale and height and CAH are highly affecting fungal growth, with the presence of high and multiscale topography features possibly disrupting the formation of stable fungal biofilms or the distribution of nutrients. Another interesting observation is that moisture is also observed at the lid of the plates that contain the superhydrophilic surfaces, an indication that the fungus is active and growing. The initial surface-attached spores became filamentous and persisted with their cyclic hyphal spore secretion. Once spherical fungal spores attach to a surface, they exhibit germ tube development, hyphal growth, and robust biofilm formation.^3^ Therefore, there is a significant increase of the TDW at 2.9 mg (2.6 times higher fungal biomass compared to the untreated PMMA surface and 16 times higher than the initial biomass) when 6 min superhydrophilic surfaces are used.

Interestingly, surface chemistry by itself also affects fungal proliferation and flat hydrophobic PMMA surfaces also slightly enhanced fungal growth (TDW = 1.46 mg), which is 1.3 times higher biomass TDW compared to the untreated ones, which are hydrophilic with WSCA 65°. Possibly, the surface chemistry of this surface containing mainly CF_*x*_ groups (*x* = 1, 2, 3) due to the plasma processing gas used for the hydrophobization of the surface is acting beneficial for fungal proliferation in comparison with untreated PMMA. It can be therefore concluded that flat surfaces with intermediate wetting properties will not exhibit strong antifungal properties, and surface chemistry and adhesion (macroscopically evaluated by CAH measurements) are a critical factor for fungal proliferation on flat as well as rough surfaces with micro/nanostructures. Interestingly, WSCA by itself is not significantly affecting fungal growth, but the synergistic effect of topography and surface chemistry, which can provide surfaces with low and high hysteresis, which is the metric for adhesion, can decrease or promote the growth of the fungus.

After comparing the results of the fungal biomass TDW for the two types of materials tested (Al and PMMA), one can easily observe that Al even when it is untreated exhibits lower biomass TDW compared to PMMA. This can be explained by taking into consideration that Al has been reported to act opposite to mycelial growth.^35−38^ Aluminum has been reported to be able to release metal ions (Al^3+^) under an acidic environment (commonly observed under the fungal proliferation period); these ions are toxic for fungi when absorbed, preventing the growth of the fungus and limiting its biomass.^39^

It is also demonstrated that if Al is transformed to superhydrophobic, the most effective antifungal concept is realized, almost eliminating fungal proliferation (0.30 ± 0.05 mg). Thus, the combination of the appropriate material choice and topography and chemistry characteristics (high and probably multiscale topography and low hysteresis) is essential when designing a “passive” antifungal surface. The interplay among roughness, wetting behavior, and fungal morphology can lead to complex effects on fungal proliferation. In the case of the superhydrophilic Al surface, the mechanical barrier imposed by surface roughness, combined with the rapid wetting dynamics, resulted in the observed decrease in TDW.

Another interesting observation is that all the superhydrophobic surfaces exhibited excellent antiadhesive performance. When washing all the surfaces with water, all spores or hyphae from the superhydrophobic surfaces were washed away easily, so they all became immediately free of fungi leftovers (see Supporting Information Section S2). On the contrary, untreated, superhydrophilic, and hydrophobic surfaces have several spores or hyphae which could not be removed, indicating their strong attachment on the surfaces (see Supporting Information Section S2), a fact that also confirms the fungal TDW increase shown in Figure 4.

### 3.3 Fungal Inhibition Zone

**Figure 5:**
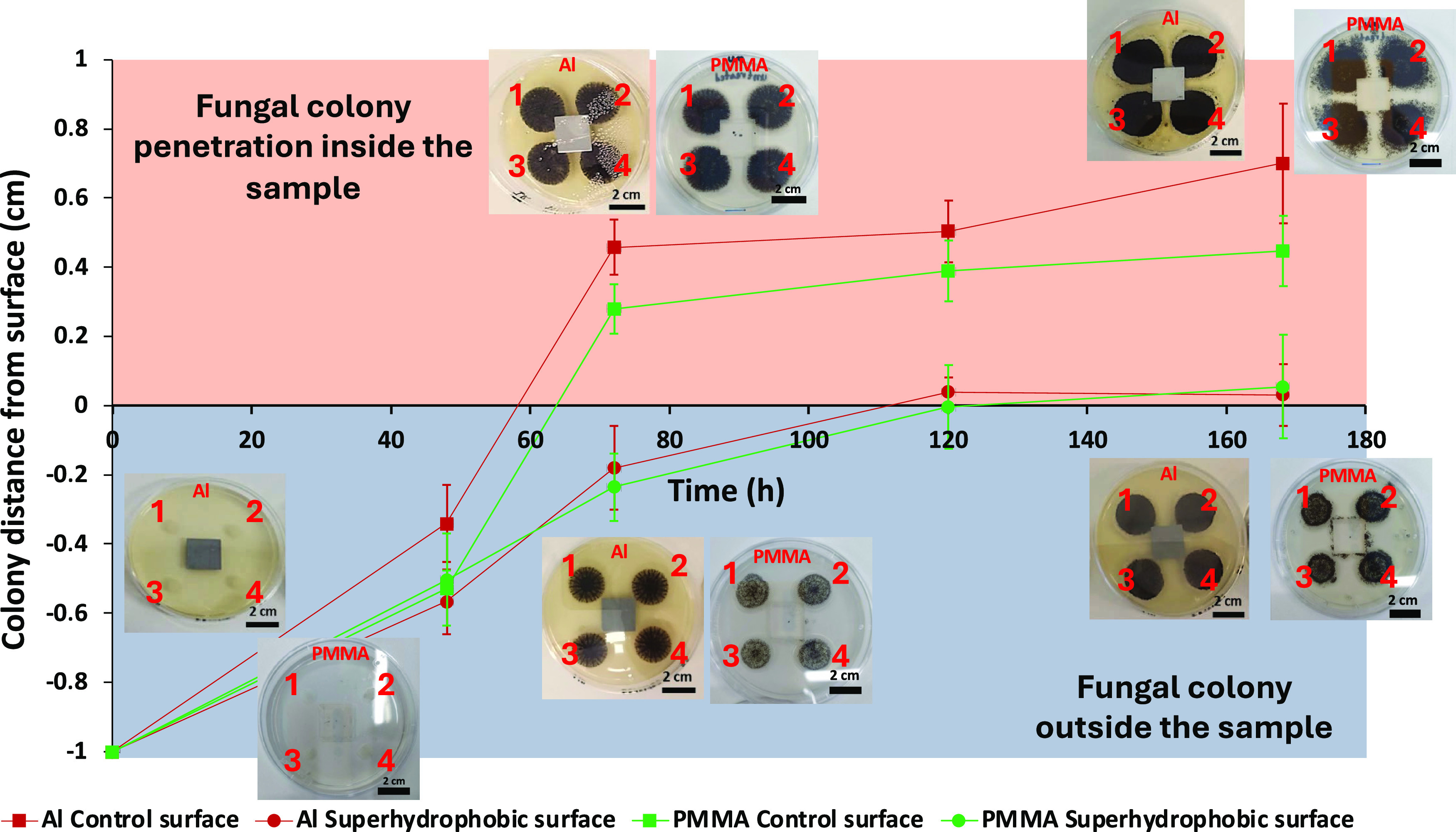
Distance of the *A. awamori* colony from untreated and superhydrophobic Al and PMMA surfaces. We consider the boundary of the surface as zero and when the fungus has not reached the surface, measurements have a minus (−) sign, while when fungus has penetrated the surface outline plus (+) sign is used. The four inoculations around the sample are marked with 1, 2, 3, and 4. On the superhydrophobic Al and PMMA surfaces, *A. awamori* is not penetrating the surface even after 168 h (7 days), whereas on the untreated (control) Al and PMMA samples, *A. awamori* penetrates inside the sample area from the first 72 h (3 days).

The antifungal properties of the superhydrophobic surfaces that exhibited the lowest TDW value from all surfaces tested are also probed by a second protocol, which involves the evaluation of the development of the fungus *A. awamori* near the surface when placed on agar containing Petri dishes. After 7 days (168 h) of incubation, the Petri dishes containing the superhydrophobic Al (12 min hierarchical with micro/nano topography) and PMMA (6 min O_2_ plasma + hydrophobic coating) surfaces showed significantly slower growth of the fungus and an “inhibition” zone around the surface was also observed, compared to the untreated Al in which the fungus penetrated inside the surface outline as well as in the untreated and PMMA surfaces (all the experiment images are provided in Section S3 of the Supporting Information). In Figure 5, we can see the results from image analysis in both untreated and superhydrophobic surfaces.

Our results indicate that starting from fungal colonies which are inoculated 1 cm away from the sample, they gradually reach and penetrate the surface area of untreated Al and PMMA samples within 72 h, while in the superhydrophobic surfaces, this does not happen even after 168 h (7 days). The fact that the fungus does not penetrate on the surface (i.e., colonies 1 and 2 are on the edge of the sample and colonies 3 and 4 are approximately 0.03 cm away from the superhydrophobic Al surface) highlights the highly effective antifungal properties of the superhydrophobic Al surfaces and the creation of an “inhibition” zone. This inhibition zone is probably created by the synergy of the material properties, with metal ions released from the surface, and the strong superhydrophobic properties, of the surface (i.e., the micro- and nanoroughness and the low surface energy serve as a mechanical barrier against the expansion of the fungus). In particular, it is commonly accepted that metal ions can disrupt essential cellular processes in fungi such as enzyme activity and protein synthesis, leading to growth inhibition. Additionally, they may induce oxidative stress within fungal cells, damaging cellular components and thus inhibiting growth. Moreover, metal ions can interfere with the integrity of fungal cell membranes, compromising their permeability.^40−43^ Last but not least, in the superhydrophobic PMMA surfaces, the fungus does not penetrate the surface similarly to Al, but some fungal spores can be observed around the sample as there is no effect such as metal ions to affect its development, so this also constitutes evidence that the choice of the material plays a crucial part in the control of fungal proliferation.

## 4 Conclusions

A complete investigation of the effect, as well as the synergy among different factors, namely, surface micro/nanotopography, material, and wettability on the proliferation control of the fungus *A. awamori* is presented. The evaluation was done using two protocols which probe different factors. Using the first protocol (under dynamic conditions), which is performed by immersing the surfaces inside a fungus spore-containing solution, it is demonstrated that superhydrophobic surfaces of both materials tested (aluminum and PMMA) significantly reduced the production of fungal TDW compared to untreated surfaces. On the other hand, micro/nanotextured superhydrophilic PMMA surfaces significantly increased the fungal proliferation resulting in 2.6 times higher fungal TDW. In addition to the aforementioned results, superhydrophobic surfaces of both materials showed excellent antifouling as well as antiadhesive properties after being washed with water. The second protocol (indirect method) is dedicated to study the growth of *A. awamori* when tested inside agar plates using conditions that normally favor fungal proliferation. These experiments revealed that superhydrophobic surfaces created an “inhibition” zone against *A. awamori*, but again, some spores were observed around the PMMA sample as there is no effect such as metal ions to affect its development. In conclusion, to realize a truly “passive” antifungal concept, (a) an appropriate material choice should be made and our data show that metals (in our case, Al) are more suitable for such applications and (b) this material should be transformed to superhydrophobic with low hysteresis (<10° or even <5°) and high multiscale topography features (several microns deep and covered with nanoscale features). The results of this study can pave the road for the integration of such functional surfaces in critical applications (i.e., food storage, medical equipment protection) in which antifungal properties are required or in fermentation applications in which accelerated fungal growth is necessary in order to increase the production yield.

## Supporting Information Available

Images of fungal growth on aluminum and PMMA surfaces that exhibit different wetting properties and micro/nanotopography, images of fungal adhesion on surfaces (after rinsing with water) and images of creation of a fungal inhibition zone (PDF)

## References

[ref1] Hawksworth D. L.; Lücking R. Fungal Diversity Revisited: 2.2 to 3.8 Million Species. Microbiol. Spectrum 2017, 5 ((4)), . 10.1128/microbiolspec.funk-0052-2016. PMC11687528 28752818

[ref2] Rosenzweig W. D.; Minnigh H.; Pipes W. O. Fungi in Potable Water Distribution Systems. J. AWWA 1986, 78 (1), 53–55. 10.1002/j.1551-8833.1986.tb05678.x.

[ref3] Rosenzweig R.; Marshall M.; Parivar A.; Ly V. K.; Pearlman E.; Yee A. F. Biomimetic Nanopillared Surfaces Inhibit Drug Resistant Filamentous Fungal Growth. ACS Appl. Bio Mater. 2019, 2 (8), 3159–3163. 10.1021/acsabm.9b00290. 35030760

[ref4] Mareković I. What’s New in Prevention of Invasive Fungal Diseases during Hospital Construction and Renovation Work: An Overview. J. Fungi 2023, 9 (2), 151 10.3390/jof9020151. PMC9966904 36836266

[ref5] World Food Programme 5 Facts about Food Waste and Hunger, 2022 https://www.wfp.org/stories/5-facts-about-food-waste-and-hunger. (accessed February 22, 2024).

[ref6] Beech I. B.; Sunner J. Biocorrosion: Towards Understanding Interactions between Biofilms and Metals. Curr. Opin. Biotechnol. 2004, 15 (3), 181–186. 10.1016/j.copbio.2004.05.001. 15193324

[ref7] Gómez-Ortíz N.; De la Rosa-García S.; González-Gómez W.; Soria-Castro M.; Quintana P.; Oskam G.; Ortega-Morales B. Antifungal Coatings Based on Ca(OH) _2_ Mixed with ZnO/TiO _2_ Nanomaterials for Protection of Limestone Monuments. ACS Appl. Mater. Interfaces 2013, 5 (5), 1556–1565. 10.1021/am302783h. 23347459

[ref8] Mitrogiannopoulou A.-M.; Tselepi V.; Ellinas K. Polymeric and Paper-Based Lab-on-a-Chip Devices in Food Safety: A Review. Micromachines 2023, 14 (5), 986 10.3390/mi14050986. 37241610 PMC10223399

[ref9] Sarkiris P.; Ellinas K.; Gkiolas D.; Mathioulakis D.; Gogolides E. Motion of Drops with Different Viscosities on Micro-Nanotextured Surfaces of Varying Topography and Wetting Properties. Adv. Funct Mater. 2019, 29 (35), 1902905 10.1002/adfm.201902905.

[ref10] Ellinas K.; Gogolides E. Ultra-Low Friction, Superhydrophobic, Plasma Micro-Nanotextured Fluorinated Ethylene Propylene (FEP) Surfaces. Micro Nano Eng. 2022, 14, 100104 10.1016/j.mne.2022.100104.

[ref11] Ioannou D.; Shah P.; Ellinas K.; Kappl M.; Sapalidis A.; Butt H.-J.; Gogolides E. Antifouling Plasma-Treated Membranes with Stable Superhydrophobic Properties for Membrane Distillation. ACS Appl. Polym. Mater. 2023, 5 (12), 9785–9795. 10.1021/acsapm.3c01512.

[ref12] Dragatogiannis D. A.; Koumoulos E.; Ellinas K.; Tserepi A.; Gogolides E.; Charitidis C. A. Nanoscale Mechanical and Tribological Properties of Plasma Nanotextured COP Surfaces with Hydrophobic Coatings. Plasma Processes Polym. 2015, 12 (11), 1271–1283. 10.1002/ppap.201500023.

[ref13] Dimitrakellis P.; Ellinas K.; Kaprou G. D.; Mastellos D. C.; Tserepi A.; Gogolides E. Bactericidal Action of Smooth and Plasma Micro-Nanotextured Polymeric Surfaces with Varying Wettability, Enhanced by Incorporation of a Biocidal Agent. Macromol. Mater. Eng. 2021, 306 (4), 2000694 10.1002/mame.202000694.

[ref14] Lee J.-W.; Hwang W. Fabrication of a Superhydrophobic Surface with Fungus-Cleaning Properties on Brazed Aluminum for Industrial Application in Heat Exchangers. Appl. Surf. Sci. 2018, 442, 461–466. 10.1016/j.apsusc.2018.02.170.

[ref15] Kim Y.; Hwang W. Wettability Modified Aluminum Surface for a Potential Antifungal Surface. Mater. Lett. 2015, 161, 234–239. 10.1016/j.matlet.2015.08.103.

[ref16] Kapica R.; Markiewicz J.; Tyczkowska-Sieroń E.; Fronczak M.; Balcerzak J.; Sielski J.; Tyczkowski J. Artificial Superhydrophobic and Antifungal Surface on Goose Down by Cold Plasma Treatment. Coatings 2020, 10 (9), 904 10.3390/coatings10090904.

[ref17] Ellinas K.; Tserepi A.; Gogolides E. Durable Superhydrophobic and Superamphiphobic Polymeric Surfaces and Their Applications: A Review. Adv. Colloid Interface Sci. 2017, 250, 132–157. 10.1016/j.cis.2017.09.003. 29021097

[ref18] Hasan J.; Crawford R. J.; Ivanova E. P. Antibacterial Surfaces: The Quest for a New Generation of Biomaterials. Trends Biotechnol. 2013, 31 (5), 295–304. 10.1016/j.tibtech.2013.01.017. 23434154

[ref19] Hashem A. H.; Saied E.; Amin B. H.; Alotibi F. O.; Al-Askar A. A.; Arishi A. A.; Elkady F. M.; Elbahnasawy M. A. Antifungal Activity of Biosynthesized Silver Nanoparticles (AgNPs) against Aspergilli Causing Aspergillosis: Ultrastructure Study. J. Funct. Biomater. 2022, 13 (4), 242 10.3390/jfb13040242. 36412883 PMC9680418

[ref20] Benkovičová M.; Kisová Z.; Bučková M.; Majková E.; Šiffalovič P.; Pangallo D. The Antifungal Properties of Super-Hydrophobic Nanoparticles and Essential Oils on Different Material Surfaces. Coatings 2019, 9 (3), 176 10.3390/coatings9030176.

[ref21] Lin N.; Berton P.; Moraes C.; Rogers R. D.; Tufenkji N. Nanodarts, Nanoblades, and Nanospikes: Mechano-Bactericidal Nanostructures and Where to Find Them. Adv. Colloid Interface Sci. 2018, 252, 55–68. 10.1016/j.cis.2017.12.007. 29317019

[ref22] Linklater D. P.; Ivanova E. P. Nanostructured Antibacterial Surfaces – What Can Be Achieved?. Nano Today 2022, 43, 101404 10.1016/j.nantod.2022.101404.

[ref23] Ellinas K.; Kefallinou D.; Stamatakis K.; Gogolides E.; Tserepi A. Is There a Threshold in the Antibacterial Action of Superhydrophobic Surfaces?. ACS Appl. Mater. Interfaces 2017, 9 (45), 39781–39789. 10.1021/acsami.7b11402. 29058866

[ref24] Kefallinou D.; Ellinas K.; Speliotis T.; Stamatakis K.; Gogolides E.; Tserepi A. Optimization of Antibacterial Properties of “Hybrid” Metal-Sputtered Superhydrophobic Surfaces. Coatings 2020, 10 (1), 25 10.3390/coatings10010025.

[ref25] Sarkiris P.; Constantoudis V.; Ellinas K.; Lam C. W. E.; Milionis A.; Anagnostopoulos J.; Poulikakos D.; Gogolides E. Topography Optimization for Sustainable Dropwise Condensation: The Critical Role of Correlation Length. Adv. Funct. Mater. 2024, 34 (1), 2306756 10.1002/adfm.202306756.

[ref26] Jafari R.; Farzaneh M. Fabrication of Superhydrophobic Nanostructured Surface on Aluminum Alloy. Appl. Phys. A 2011, 102 (1), 195–199. 10.1007/s00339-010-6131-0.

[ref27] Ellinas K.; Pujari S. P.; Dragatogiannis D. A.; Charitidis C. A.; Tserepi A.; Zuilhof H.; Gogolides E. Plasma Micro-Nanotextured, Scratch, Water and Hexadecane Resistant, Superhydrophobic, and Superamphiphobic Polymeric Surfaces with Perfluorinated Monolayers. ACS Appl. Mater. Interfaces 2014, 6 (9), 6510–6524. 10.1021/am5000432. 24749933

[ref28] Ellinas K.; Tserepi A.; Gogolides E. From Superamphiphobic to Amphiphilic Polymeric Surfaces with Ordered Hierarchical Roughness Fabricated with Colloidal Lithography and Plasma Nanotexturing. Langmuir 2011, 27 (7), 3960–3969. 10.1021/la104481p. 21351799

[ref29] Nioras D.; Ellinas K.; Gogolides E. Atmospheric Water Harvesting on Micro-Nanotextured Biphilic Surfaces. ACS Appl. Nano Mater. 2022, 5 (8), 11334–11341. 10.1021/acsanm.2c02439.

[ref30] Tzianou M.; Thomopoulos G.; Vourdas N.; Ellinas K.; Gogolides E. Tailoring Wetting Properties at Extremes States to Obtain Antifogging Functionality. Adv. Funct. Mater. 2021, 31 (1), 2006687 10.1002/adfm.202006687.

[ref31] Tsougeni K.; Petrou P. S.; Awsiuk K.; Marzec M. M.; Ioannidis N.; Petrouleas V.; Tserepi A.; Kakabakos S. E.; Gogolides E. Direct Covalent Biomolecule Immobilization on Plasma-Nanotextured Chemically Stable Substrates. ACS Appl. Mater. Interfaces 2015, 7 (27), 14670–14681. 10.1021/acsami.5b01754. 26098201

[ref32] Filippou I.; Tselepi V.; Ellinas K. A Review of Microfabrication Approaches for the Development of Thin, Flattened Heat Pipes and Vapor Chambers for Passive Electronic Cooling Applications. Micro Nano Eng. 2024, 22, 100235 10.1016/j.mne.2023.100235.

[ref33] Ellinas K.; Pliaka V.; Kanakaris G.; Tserepi A.; Alexopoulos L. G.; Gogolides E. Micro-Bead Immunoassays for the Detection of IL6 and PDGF-2 Proteins on a Microfluidic Platform, Incorporating Superhydrophobic Passive Valves. Microelectron. Eng. 2017, 175, 73–80. 10.1016/j.mee.2017.02.015.

[ref34] Taniwaki M. H.; Pitt J. I.; Magan N. Aspergillus Species and Mycotoxins: Occurrence and Importance in Major Food Commodities. Curr. Opin. Food Sci. 2018, 23, 38–43. 10.1016/j.cofs.2018.05.008.

[ref35] Lewis J. A. Effect of Mineral Salts on Aphanomyces Euteiches and Aphanomyces Root Rot of Peas. Phytopathology 1973, 63 (8), 989 10.1094/Phyto-63-989.

[ref36] Smith W. H.; Staskawicz B. J.; Harkov R. S. Trace-Metal Pollutants and Urban-Tree Leaf Pathogens. Trans. Br. Mycol. Soc. 1978, 70 (1), 29–33. 10.1016/S0007-1536(78)80166-1.

[ref37] Thompson G. W.; Medve R. J. Effects of Aluminum and Manganese on the Growth of Ectomycorrhizal Fungi. Appl. Environ. Microbiol. 1984, 48 (3), 556–560. 10.1128/aem.48.3.556-560.1984. 16346623 PMC241565

[ref38] Piña R. G.; Cervantes C. Microbial Interactions with Aluminium. BioMetals 1996, 9 (3), 311–316. 10.1007/BF00817932. 8696081

[ref39] He G.; Wang X.; Liao G.; Huang S.; Wu J. Isolation, Identification and Characterization of Two Aluminum-Tolerant Fungi from Acidic Red Soil. Indian J. Microbiol. 2016, 56 (3), 344–352. 10.1007/s12088-016-0586-4. 27407299 PMC4920765

[ref40] Slavin Y. N.; Bach H. Mechanisms of Antifungal Properties of Metal Nanoparticles. Nanomaterials 2022, 12 (24), 4470 10.3390/nano12244470. 36558323 PMC9781740

[ref41] Mancier V.; Fattoum S.; Haguet H.; Laloy J.; Maillet C.; Gangloff S. C.; Chopart J.-P. Antifungal and Coagulation Properties of a Copper (I) Oxide Nanopowder Produced by Out-of-Phase Pulsed Sonoelectrochemistry. Antibiotics 2024, 13 (3), 286 10.3390/antibiotics13030286. 38534722 PMC10967388

[ref42] Babele P. K.; Singh A. K.; Srivastava A. Bio-Inspired Silver Nanoparticles Impose Metabolic and Epigenetic Toxicity to Saccharomyces Cerevisiae. Front. Pharmacol. 2019, 10, 466706 10.3389/fphar.2019.01016. PMC6751407 31572189

[ref43] Priyadarshini E.; Priyadarshini S. S.; Cousins B. G.; Pradhan N. Metal-Fungus Interaction: Review on Cellular Processes Underlying Heavy Metal Detoxification and Synthesis of Metal Nanoparticles. Chemosphere 2021, 274, 129976 10.1016/j.chemosphere.2021.129976. 33979913

