# Supplementary material for: Functional Surfaces for Passive Fungal Proliferation Control: Effect of Surface Micro- and Nanotopography, Material, and Wetting Properties
Source: ACS Appl Bio Mater. 2024 Jul 1;7(7):4510–8. doi: 10.1021/acsabm.4c00387 (PMC11253093; doi:10.1021/acsabm.4c00387)
Supplement: Supplementary file 1 — mt4c00387_si_001.pdf [file mt4c00387_si_001.pdf]

Supporting Information

Functional surfaces for passive fungal proliferation control: Effect of surface micro and nano topography, material, and wetting properties

Vasiliki Tselepi[1], Panagiotis Sarkiris[2], Dimitrios Nioras[2], Erminta Tsouko[3,4], Dimitrios Sarris[3], Evangelos Gogolides[2], Kosmas Ellinas[1]*

[1]Laboratory of Advanced Functional Materials and Nanotechnology, Department of Food Science and Nutrition, School of the Environment, University of the Aegean, Leoforos Dimokratias 66, Myrina 81400, Lemnos, Greece

[2] Institute of Nanoscience and Nanotechnology NCSR "Demokritos", 15341 Aghia Paraskevi, Attiki, Greece

[3]Laboratory of Physico-Chemical and Biotechnological Valorization of Food By-Products, Department of Food Science & Nutrition, School of Environment, University of the Aegean, Leoforos Dimokratias 66, Myrina 81400, Lemnos, Greece

[4]Theoretical and Physical Chemistry Institute, National Hellenic Research Foundation, 48 Vassileos Constantinou Ave., 11635, Athens, Greece

Corresponding author: kellinas@aegean.gr

S1. Fungal growth/inhibition on Al and PMMA surfaces exhibiting different wetting properties and micro-nanotopography

Images of the growth of the fungus *Aspergillus awamori* were captured to monitor fungal growth on the different surfaces. Below, we present some indicative images of the fungal development.

- Stereoscope images of Aluminum surfaces

1 week	2 weeks
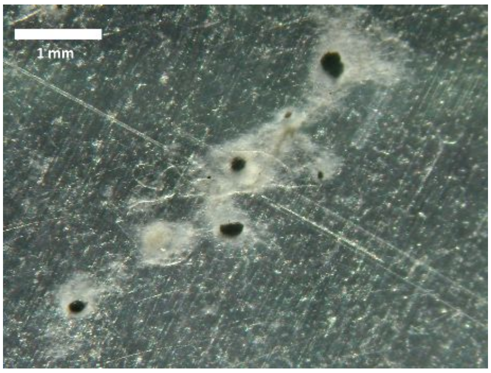	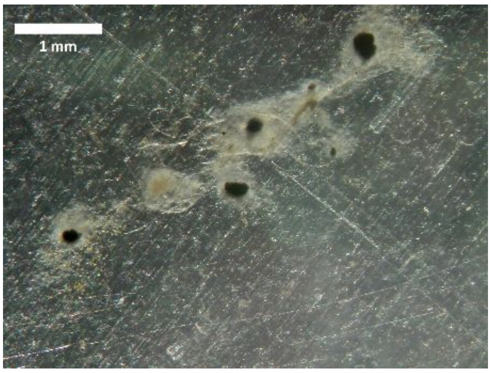
(a)	(b)
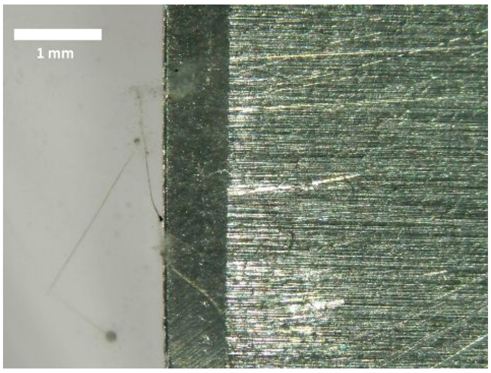	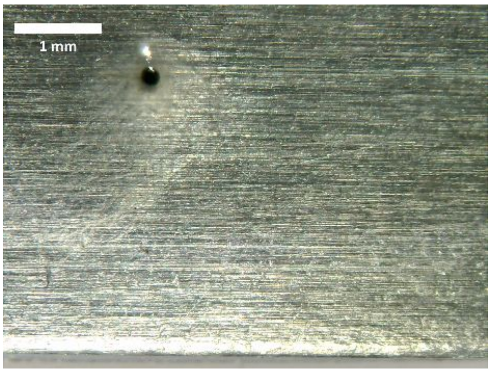
(c)	(d)
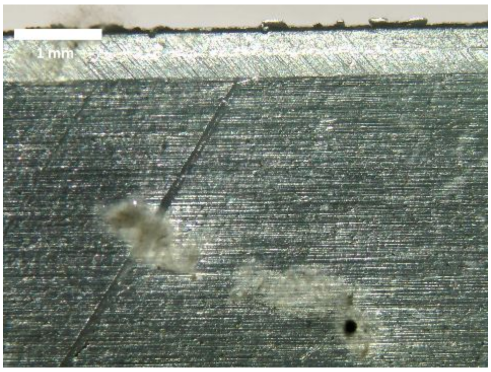	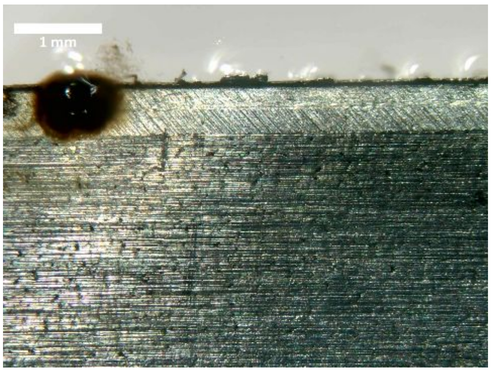

(e)	(f)
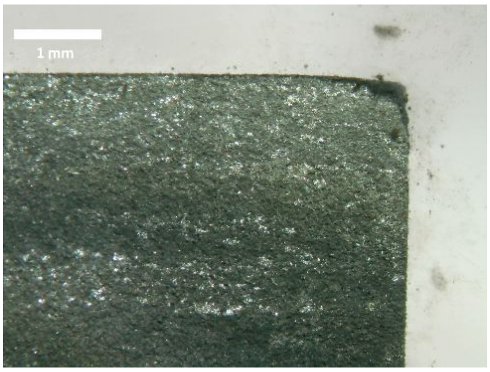	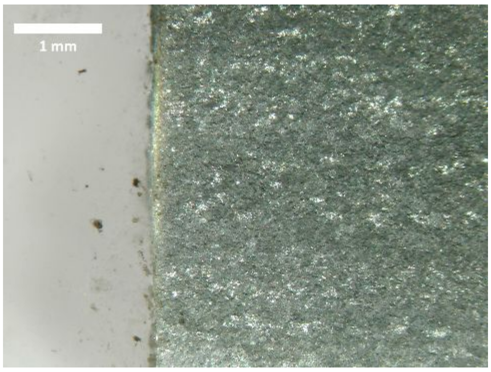
(g)	(h)

Figure S1. Development of *Aspergillus awamori* for 2 weeks, inside the 6-well plate with the Aluminum surfaces. Images in 1- and 2-weeks duration were taken with a stereoscope equipped with a camera. (a), (b) untreated surfaces, (c), (d) superhydrophilic surfaces, (e), (f) hydrophobic flat surfaces + C_4F_8, (g), (h) superhydrophobic surfaces. It is noted that in all the plates that contain the aluminum surfaces, fungal hyphae are found mostly inside the suspension and not on the surfaces. The 6 min superhydrophobic surfaces exhibited the best antifouling properties with no fungal hyphae observed on them throughout the entire experiment duration.

- Stereoscope images of PMMA surfaces

**1 week**	**2 weeks**
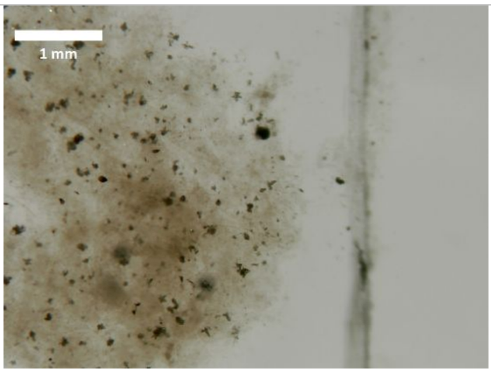	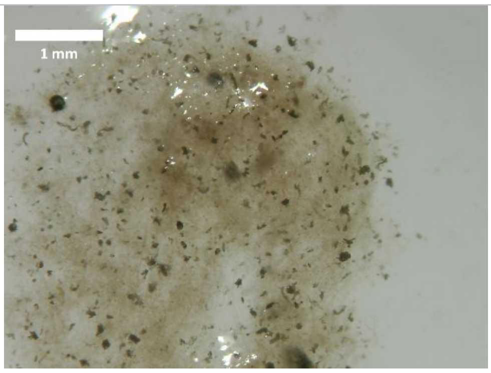
(a)	(b)

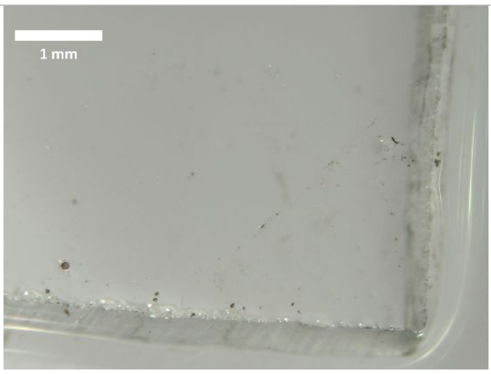

(c)

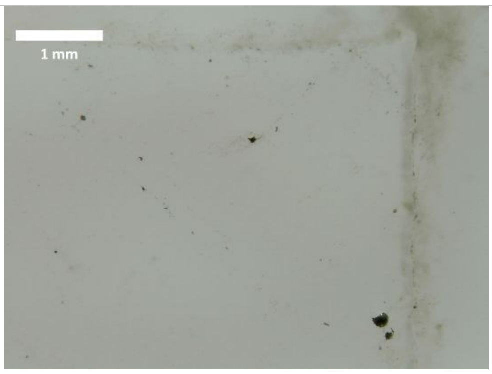

(d)

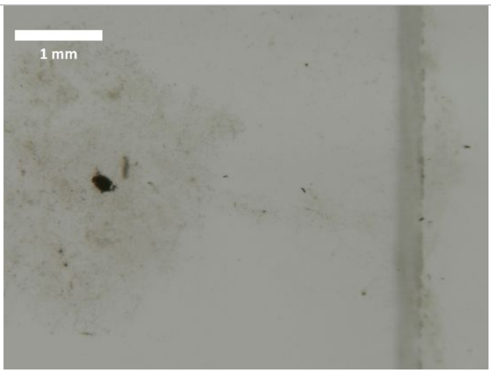

(e)

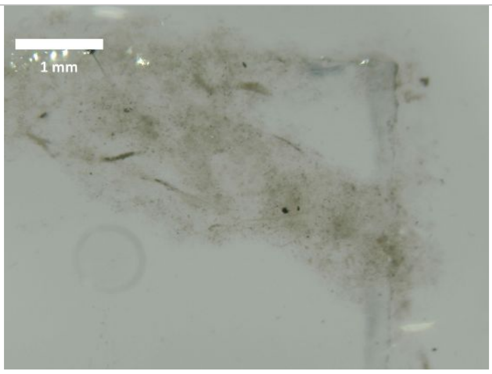

(f)

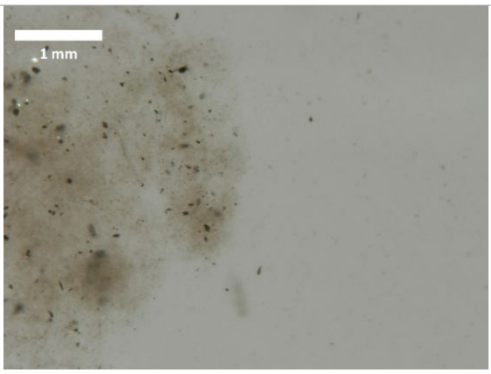

(g)

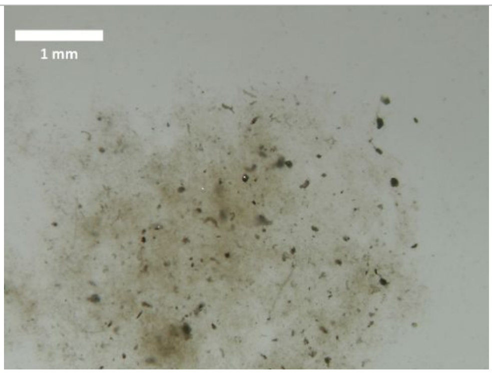

(h)

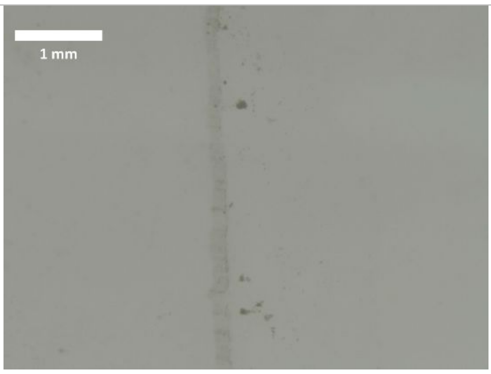

(i)

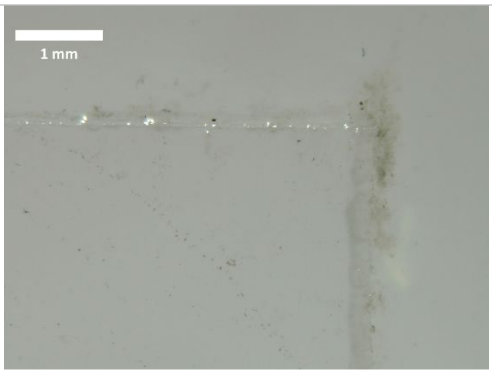

(j)

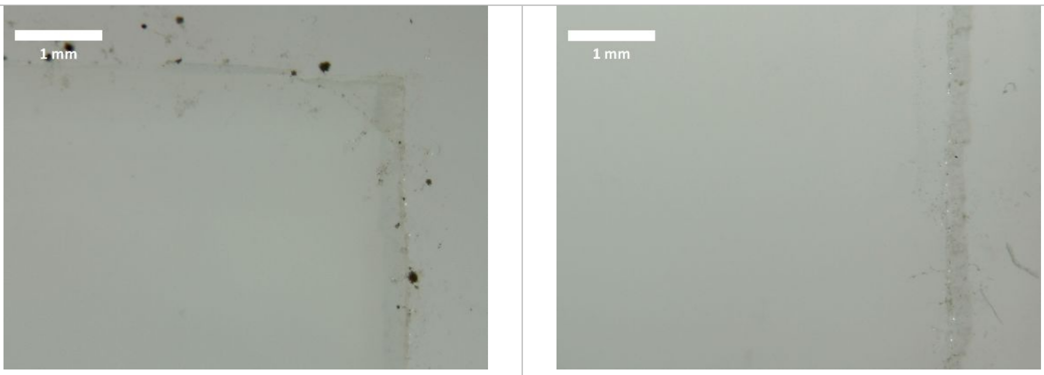

(k) (l)

Figure S2. Development of *Aspergillus awamori* for 2 weeks, inside the 6-well plate with the PMMA surfaces. Images in 1- and 2-weeks duration were taken with a stereoscope: (a)-(b) untreated surfaces, (c)-(d) hydrophobic flat surfaces with C_4F_8 coating, (e)-(f) hydrophilic surfaces with 1 min O_2 plasma treatment, (g)-(h) superhydrophilic surfaces with 6 min plasma O_2 treatment, (i)-(j) superhydrophobic surfaces with 1 min O_2 plasma treatment and C_4F_8 coating, (k)-(l) superhydrophobic surfaces with 6 min O_2 plasma treatment and C_4F_8 coating. Many spore aggregates can be seen on the untreated as well as on the superhydrophilic surfaces. On the contrary, few or none, fungal spores are observed on superhydrophobic surfaces, highlighting their antifouling properties. The images shown here are not directly related to the total biomass of each case, because the biomass TDW is calculated from the entire volume of the fungal spore suspension inside the well containing each surface.

S2. Fungal adhesion on surfaces

There is much more interest when we observe the surfaces under an optical microscope or a stereoscope to test whether the fungus has been washed away during the washing step with water. Both aluminum and PMMA superhydrophobic surfaces exhibit anti-adhesive properties deriving from the self-cleaning properties of superhydrophobicity. On the contrary, the fungus is strongly attached on the non-superhydrophobic surfaces. In the images below, the difference in the color of the surfaces comes from the texturing created on the superhydrophobic and

superhydrophilic ones, since where there is micro- and nanostructure the surfaces appears darker in the image of the optical microscope.

- Stereoscope images of Aluminum surfaces

**Aluminum**
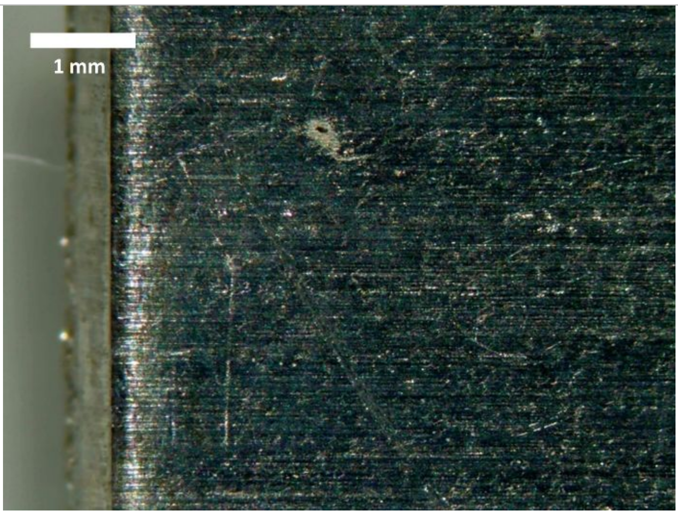<br>(a)
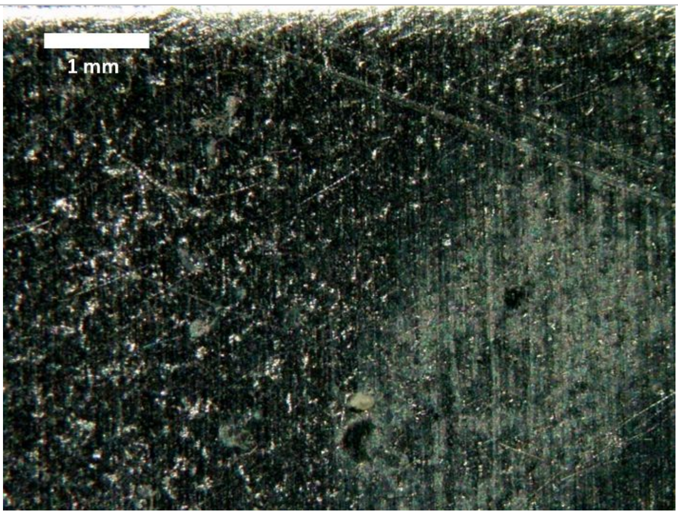<br>(b)

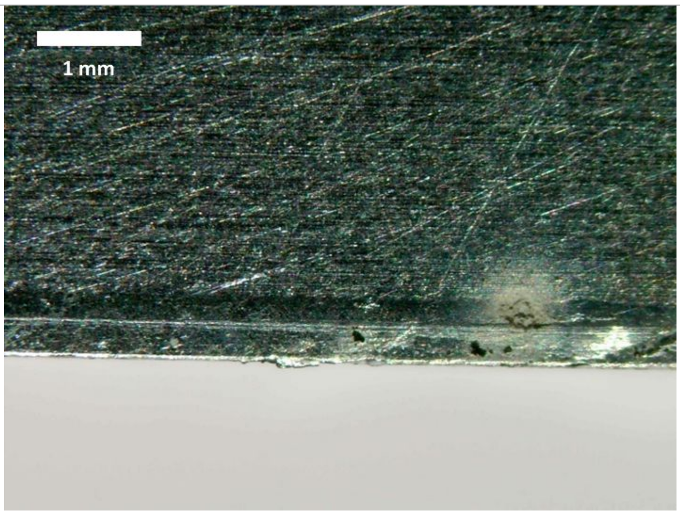

(c)

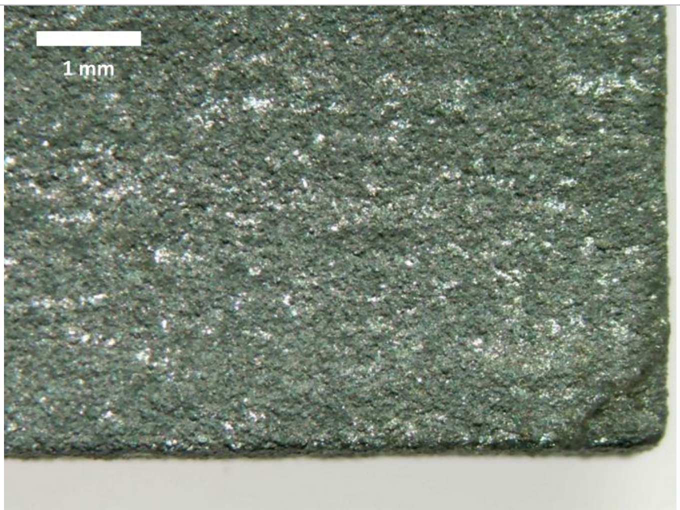

(d)

Figure S3. Adhesion of *Aspergillus awamori* on aluminum surfaces after rinsing with water. Images were taken from stereoscope (magnification 6.5x): (a), (b) untreated surfaces, (c), (d) superhydrophilic surfaces (5 min), (e), (f) hydrophobic flat surfaces + C_4F_8, (g), (h) superhydrophobic + C_4F_8 surfaces (12 min). No fungal spores remained on this type of surface, highlighting the anti-adhesive properties of the superhydrophobic Al surface.

- Images of PMMA surfaces with optical microscope

PMMA
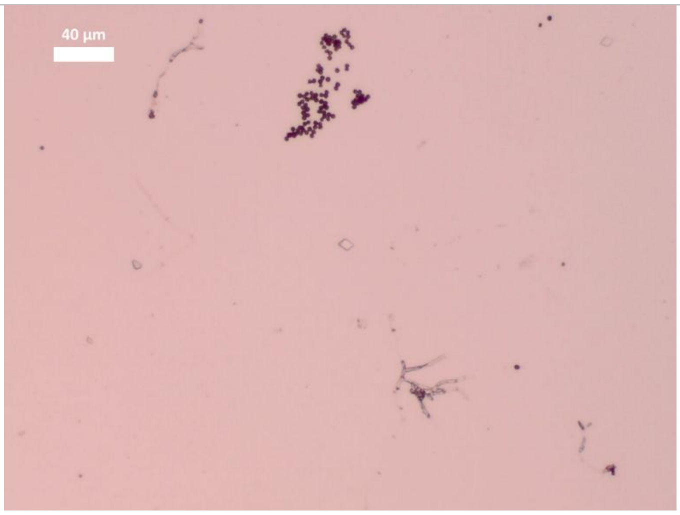 (a)
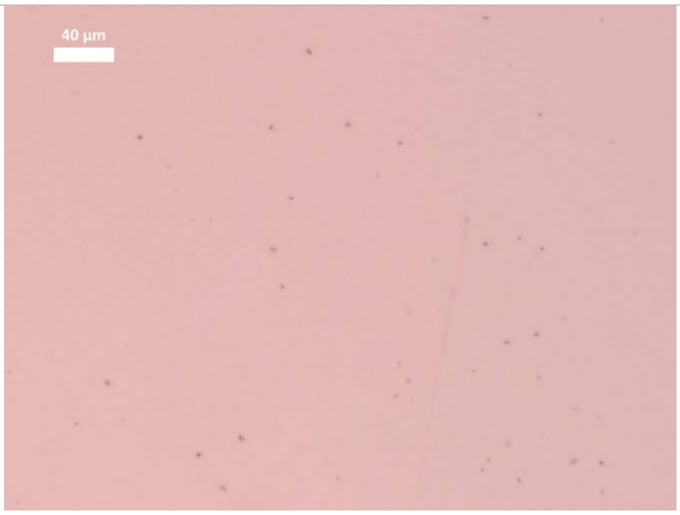 (b)
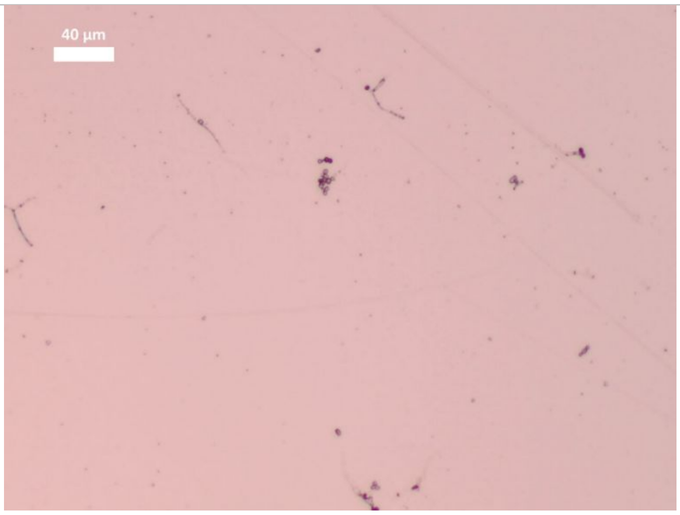 (c)

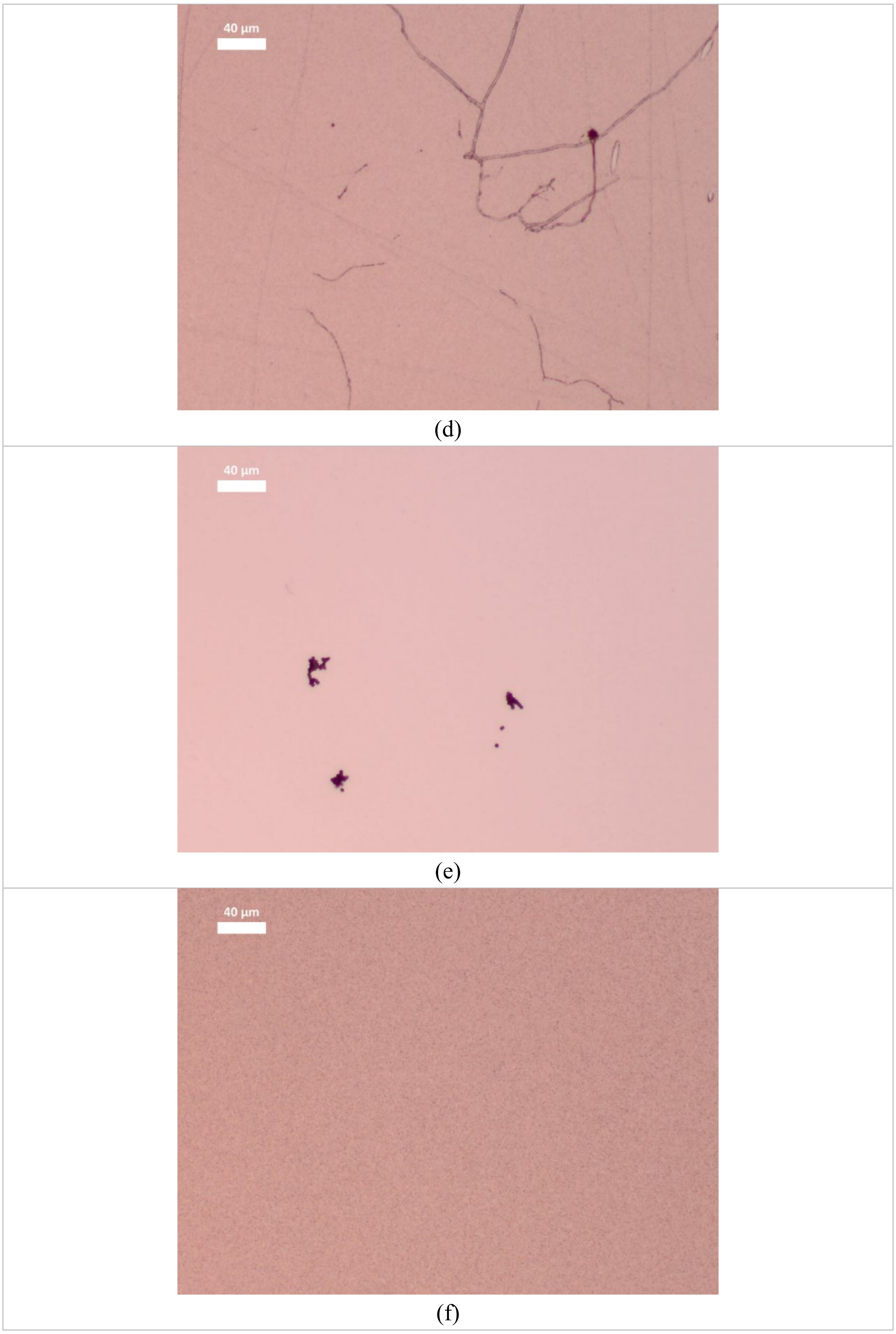

Figure S4. Adhesion of *Aspergillus awamori* on PMMA surfaces after rinsing with water. Images were taken with an optical microscope: (a) untreated surface, (b) hydrophobic flat surface with C_4F_8 coating, (c) hydrophilic surface after 1 min O_2 plasma treatment, (d)

superhydrophilic surface after 6 min O_2 plasma treatment, (e) superhydrophobic surface after 1 min O_2 plasma treatment and C_4F_8 coating, (f) superhydrophobic surface after 6 min O_2 plasma treatment and C_4F_8 coating. No fungi spores were found on the 6 min superhydrophobic surface highlighting their anti-adhesive properties.

S3. Fungal proliferation on surfaces evaluation: Creation of a fungal inhibition zone

0 h	48 h	72 h	120 h	168 h
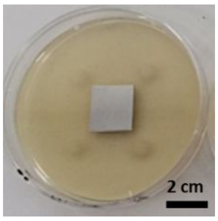	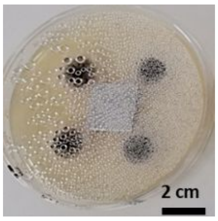	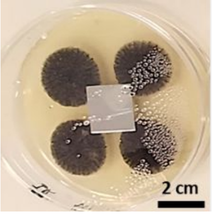	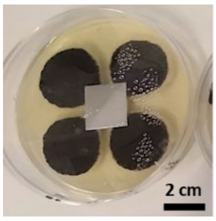	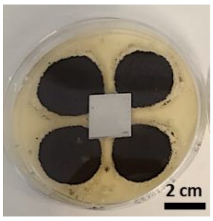
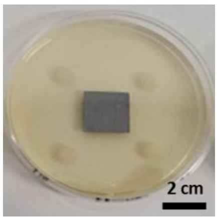	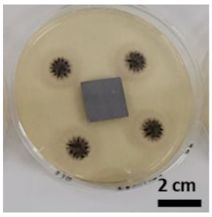	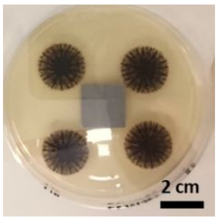	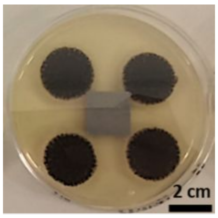	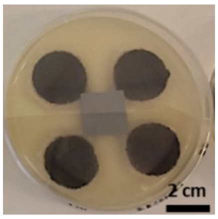

Figure S5. First row: Untreated aluminum surface used as control surface. Second row: Superhydrophobic aluminum surface. The images clearly show that on superhydrophobic Al surface the colony has not reached or overcame the surface outline borders, highlight the passive antifungal action of this type of surfaces as a result of topography with multiscale features, material, and wetting properties.

0 h	48 h	72 h	120 h	168 h
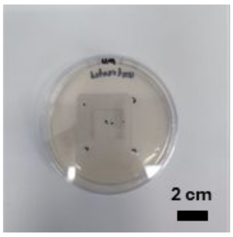	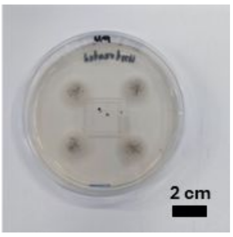	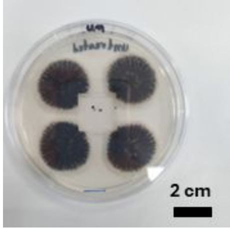	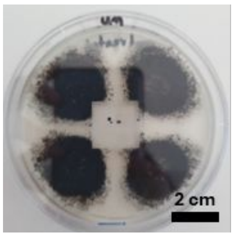	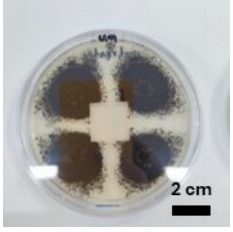

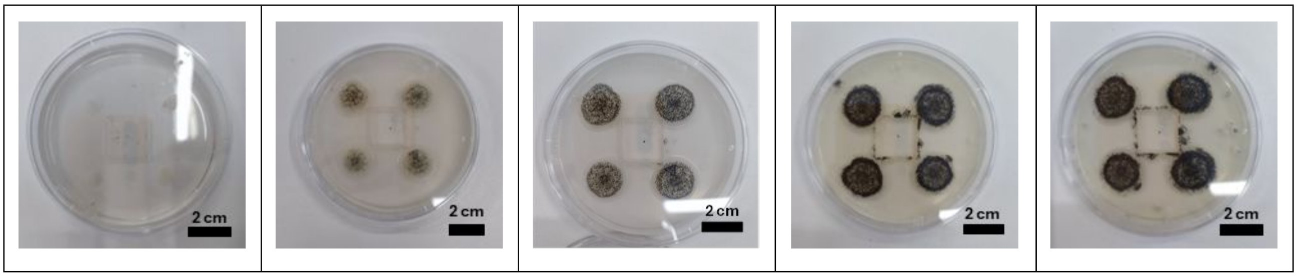

Figure S6. First row: Untreated PMMA surface used as control surface. Second row: Superhydrophobic PMMA surface. The colony has not reached or overcame the surface outline borders, highlighting the passive antifungal action of this type of surfaces as a result of topography with multiscale features, and wetting properties.
